# Alteration of blood clot structures by interleukin-1 beta in association with bone defects healing

**DOI:** 10.1038/srep35645

**Published:** 2016-10-21

**Authors:** Xin Wang, Thor E. Friis, Paul P. Masci, Ross W. Crawford, Wenbo Liao, Yin Xiao

**Affiliations:** 1 Institute of Health and Biomedical Innovation, Queensland University of Technology, Brisbane, 4059 Queensland, Australia; 2 Department of Orthopaedic Surgery, Affiliated Hospital of Zunyi Medical University, Zunyi, 563000 Guizhou, China; 3 Australia-China Centre for Tissue Engineering and Regenerative Medicine, Queensland University of Technology, Brisbane, 4059 Queensland, Australia; 4 Translational Research Institute, School of Medicine, The University of Queensland, Brisbane, 4102 Queensland, Australia

## Abstract

The quality of hematomas are crucial for successful early bone defect healing, as the structure of fibrin clots can significantly influence the infiltration of cells, necessary for bone regeneration, from adjacent tissues into the fibrin network. This study investigated if there were structural differences between hematomas from normal and delayed healing bone defects and whether such differences were linked to changes in the expression of IL-1β. Using a bone defect model in rats, we found that the hematomas in the delayed healing model had thinner fibers and denser clot structures. Moreover, IL-1β protein levels were significantly higher in the delayed healing hematomas. The effects of IL-1β on the structural properties of human whole blood clots were evaluated by thrombelastograph (TEG), scanning electronic microscopy (SEM), compressive study, and thrombolytic assays. S-nitrosoglutathione (GSNO) was applied to modulate *de novo* hematoma structure and the impact on bone healing was evaluated in the delayed healing model. We found that GSNO produced more porous hematomas with thicker fibers and resulted in significantly enhanced bone healing. This study demonstrated that IL-1β and GSNO had opposing effects on clot architecture, the structure of which plays a pivotal role in early bone healing.

Fracture hematomas (blood clot) are fibrin scaffolds that are produced immediately following a bone injury and are considered a vital element of fracture healing. Removal of the initial fracture hematoma impairs the repair process, whereas implantation of a hematoma can yield new bone formation in a rat model^1^. The healing process can be divided into three key consecutive and overlapping phases: (1) inflammation, (2) reparative, and (3) remodelling, and is affected by a number of factors^2^. Most previous studies on fracture healing have tended to focus on the late stages of the healing process. As a result, the involvement of early proinflammatory cytokines in developing fracture hematoma structures remains poorly understood. However, the chemotaxic activity of proinflammatory cytokines originating from activated platelets and immune cells is thought to be a prerequisite for cell infiltration from the broken bone segments, thus controlling bone repair process^3^,^4^.

To date, autologous platelet concentrates, such as platelet-rich plasma (PRP) have been trialled as potential bioactive materials to enhance bone regeneration. It was thought that these treatments would provide a fibrin matrix that could act as a reservoir that could release a consistent flow of growth factors^5^. However, there are studies casting doubts on the efficacy of PRP since these treatments showed little evidence of accelerating the bone repair process in either animals or humans^6^,^7^. One explanation for this lack of efficacy is the significantly denser fibrin meshwork found in PRP, which is supposed to be due to the supra-physiological levels of thrombin used. The dense fibrin clot decreases permeability and significantly retards cellular migration from adjacent tissues^8^. Such artificial *ex vivo* fibrin scaffolds have smaller pore sizes and been shown to slow bone healing in rats^9^. By contrast, porous fibrin clots composed of thicker fibers has been shown to promote migration of endothelial cells and enhance ossification within injured sites^10^. This indicates that variations in fibrin structure properties (such as pore size) can considerably modulate the bone healing process^11^.

The acute phase response (APR) serves as the body’s first response to local disturbances caused by bone trauma and is characterized by the release of proinflammatory cytokines, such as interleukin-1β (IL-1β)^12^. Concurrently, a hematoma made up of platelets and fibrin fibers forms at the bone fracture sites to minimize bleeding^13^. In the clotting process, activation and subsequent aggregation of platelets leads to the secretion of cytokines and chemokines which regulate hemostasis. This process directly influences fibrin clot architecture and affects the subsequent reparative process^14^,^15^. Elevated levels of thrombospondin from activated platelets are inversely proportional to the fiber diameter and porosity of the clots^16^.

There is ample evidence to suggest that the inflammatory reactions can promote a prothrombotic state and initiate thrombogenesis^17^. At fracture sites, the chemotaxic effect of IL-1β on mesenchymal stem cells has been implicated as a main trigger contributing to early healing within a day of a fracture^18^. Although IL-1β has been reported to bind directly to fibrinogen^19^, there are no studies that have demonstrated its direct effects on fibrin structure during hemostasis. S-nitrosoglutathione (GSNO) has been shown to modulate fibrin structure by increasing fiber thickness and has, therefore, been used to treat thrombosis^20^,^21^. In this study, we have investigated whether IL-1β exerts a regulatory function on fibrin polymerization and whether, by altering the hematoma structure with GSNO, it is possible to speed up bone regeneration in large bone defects. Altering the physical structure of the hematoma by means of biological agents such as GSNO may prove to be a valid therapeutic approach to augment recalcitrant bone fractures.

## Results

### Delayed bone healing showed differences in hematoma structure and higher level of IL-1β

**Figure 1:**
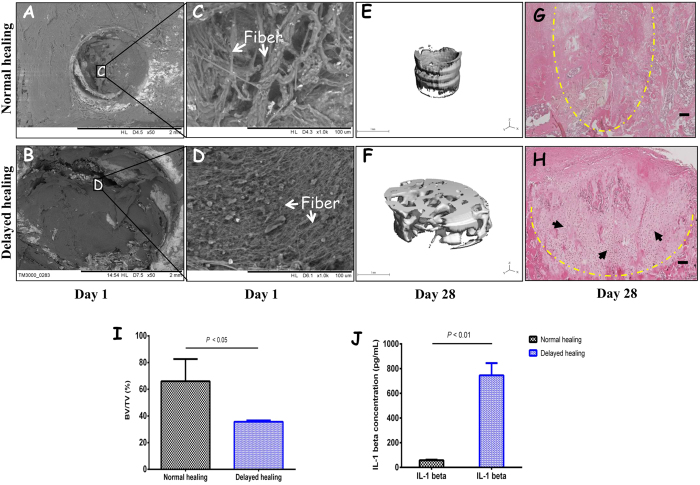
Delayed bone healing samples showed structural differences in hematomas and higher IL-1β levels. The fiber morphology of hematomas in the 1 mm (**A**,**C**) and 3 mm (**B**,**D**) defects was assessed using SEM at day 1 (scale bar = 2 mm and 100 μm, respectively) (*n* = 6). De novo bone formation was evaluated using micro-CT (**E**,**F**,**I**) (scale bar = 1 mm) and the histology was assessed by H&E staining (**G**,**H**) (scale bar = 100 μm) (*n* = 6). The amount of IL-1β protein in hematomas within the two defect sizes was measured using ELISA (*n* = 6) (**J**). Yellow dash line indicated the edge of defects and arrow heads represents cartilage.

The fibrin network of hematomas in the 1 mm defects had thicker fibers in a loose configuration (white arrow), whereas the 3 mm hematomas had thinner fibers in a tighter structure (Fig. 1A–D). Fiber diameters were measured to be 397.6 ± 126.6 nm in normal bone healing defects and 245.8 ± 41.68 nm in delayed bone healing defects (*p* < 0.01). Micro-CT analysis showed a significantly lower bone volume/total volume (BV/TV) ratio and a delayed healing process in the 3 mm defects (35.7% ± 1.0%) compared with 1 mm defect (66.0% ± 16.6%) (Fig. 1E,F,I). This was further confirmed by histology, which showed a considerable amount of unmineralized cartilage within the 3 mm defects (Fig. 1H). By contrast, in 1 mm defects there was no evidence of *in situ* cartilage (Fig. 1G). The bone healing process in the 1 mm and 3 mm diameter defect model in rats were consistent with previous studies demonstrating that 1-mm-diameter defect healed rapidly through direct healing (intramembranous ossification), while 3-mm-diameter defect healed slowly via indirect healing (endochondral ossification)^22^,^23^. Furthermore, we found that endogenous IL-1β levels were 13 times higher in the 3 mm defects compared to the 1 mm defects (745.40 ± 99.19 pg/mL vs. 57.46 ± 4.72 pg/mL; *p* < 0.01) (Fig. 1J).

### IL-1β influences clot kinetic of human blood clots

**Figure 2:**
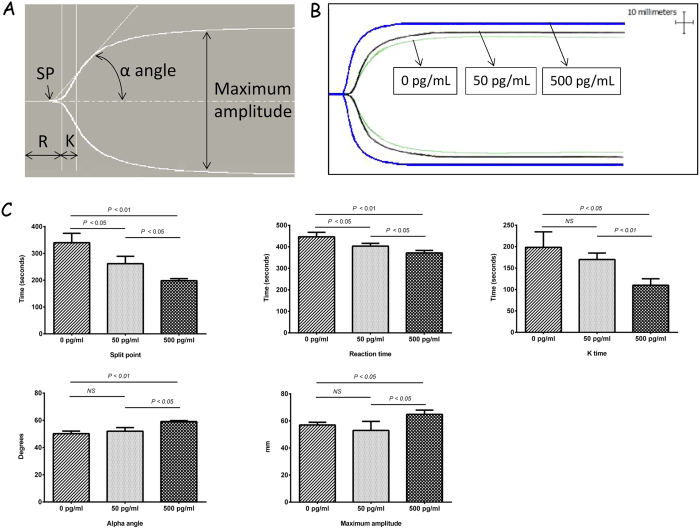
IL-1β influences thrombosis process of human blood clots. (**A**) Diagrammatic representation of a standard TEG profile demonstrating the generally described variables: SP, split point; R, reaction time; K, coagulation time; α angle; MA, maximum amplitude. (**B**) Representative TEG traces of human whole blood clots treated with 0, 50 and 500 pg/mL concentrations of IL-1β (*n* = 3). (**C**) Effects of different concentrations of IL-1β on TEG parameters of human whole blood clots. (*n* = 3, NS indicated no significant difference).

Hemostatic parameters, such as SP, R-time, K-time, alpha angle (α), and MA, were assessed using a TEG analyser. Initial fibrin formation time, reflected by SP and R, was significantly shorter in the 500 pg/mL IL-1β group compared to the control group (SP, 198.3 ± 7.6 vs. 340.0 ± 35.0, s; R time, 371.7 ± 11.6 vs. 446.7 ± 20.8, s) (Fig. 2). Thrombosis formation speed is expressed by both K time and α angle, while MA is a measure of the maximal strength of the clots. The K value was significantly shorter in the 500 pg/mL IL-1β group compared to the controls (K, 110.0 ± 15.0 vs. 198.3 ± 36.2, s), whereas the α angle and clot strength (MA values) of the whole blood clots increased noticeably in the 500 pg/mL IL-1β group compared with the control group (α, 59.1 ± 0.7 vs. 50.2 ± 1.9, degree; MA, 64.9 ± 3.1 vs. 57.0 ± 2.0, mm) (Fig. 2C).

### IL-1β influences fibrin clot structure of human blood clots

**Figure 3:**
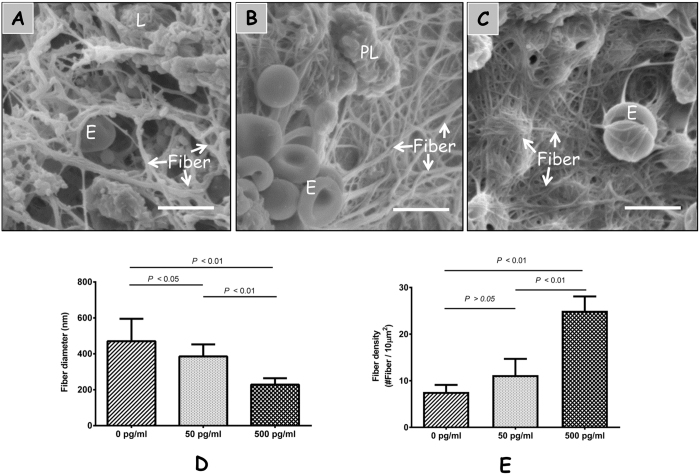
IL-1β influences fibrin construct of human blood clots. Scanning electron micrographs of blood clot networks in presence of formed by 0 (**A**), 50 (**B**), 500 (**C**) pg/mL IL-1β (scale bar = 5 μm, *n* = 3). Average diameter of fibers (*n* = 90) (**D**) and average density of fibers (**E**) were calculated using Image J software. E = erythrocytes, L = leukocytes, and PL = platelets.

Structural analysis of blood clots formed with or without IL-1β by SEM showed a significant difference in fiber thickness between the IL-1β and control group (Fig. 3A–C). In the 50 and 500 pg/mL IL-1β treatment groups, the clots exhibited significantly thinner fibers (385.6 ± 67.0 nm and 228.8 ± 35.4 nm, respectively) compared to the controls (470.4 ± 124.7 nm) (*p* < 0.05) (Fig. 3D). Clots formed in the presence of 500 pg/mL IL-1β exhibited a higher density of fibers (25 ± 3.1 fiber per 10 μm^2^) than the controls (7.6 ± 1.5 fiber per 10 μm^2^) (*p* < 0.01) (Fig. 3E). The fibrin networks in the 500 pg/mL IL-1β group had discernibly thinner and more densely packed fibers compared to the control group (Fig. 3,A,C).

### IL-1β influences biomechanical property of human blood clots

**Figure 4:**
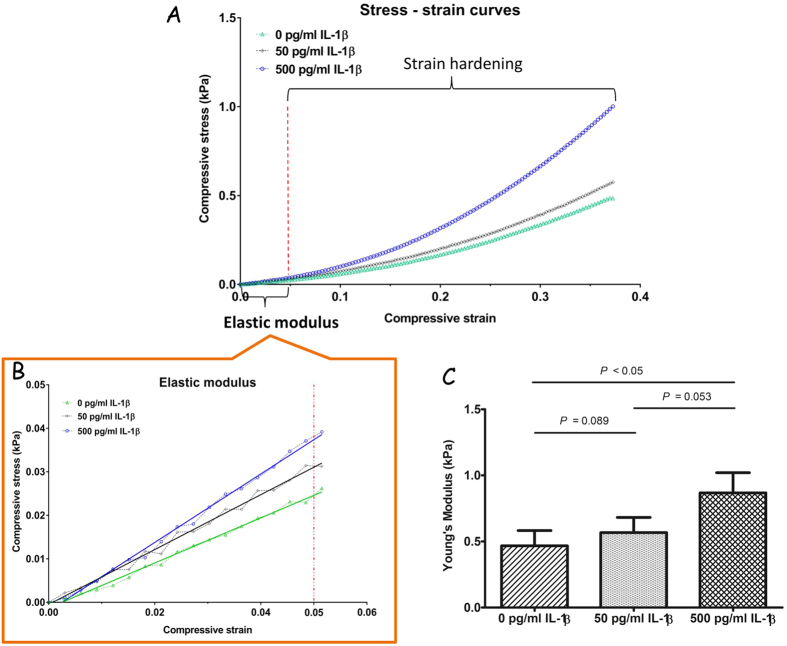
IL-1β influences biomechanical property of human blood clots. Influence of IL-1β on the mechanical properties of whole blood clots (*n* = 3). (**A**) Representative stress-strain curves plotted from compressive test on whole blood clots formed in the presence of 0, 50 and 500 pg/mL of IL-1β. (**B**,**C**) Young’s modulus was measured by calculating the slope in the initial linear proportion (0–5%) (*n* = 3) (see Methods and Materials). In addition, the Young’s modulus is not a constant but increases as the clot responds to compression, this phase is defined as strain hardening (non-linear proportion) owing to fibrin meshwork deformation of blood clots.

The thickness and diameter of blood clot formed for the mechanical test in average were 6.5 mm and 12 mm, respectively. Mechanical testing in the form of compression analysis evaluated the effect of IL-1β on mechanical properties of blood clot formation with (50 or 500 pg/mL) and without (control) IL-1β (Fig. 4A–C). There was no statistically significant difference in Young’s moduli between the control group (0.47 ± 0.12 kPa) and 50 pg/mL IL-1β group (0.57 ± 0.12 kPa) (*p* > 0.05). However, the Young’s modulus of the 500 pg/mL IL-1β treatment group at 0.87 ± 0.15 kPa was significantly higher than the controls (*p* < 0.05).

### IL-1β influences the susceptibility of human blood clots to thrombolysis

**Figure 5:**
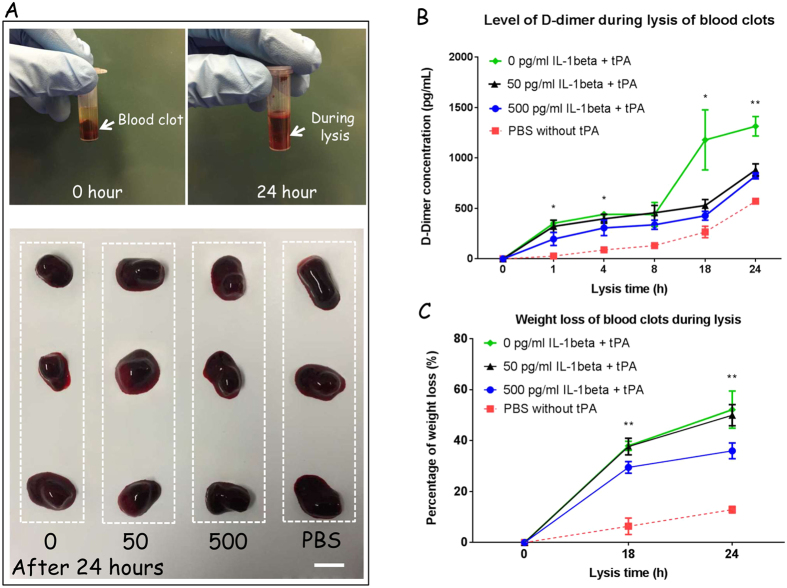
IL-1β influences the susceptibility of human blood clots to thrombolysis. Photographs of thrombolysis of whole blood clots incubated with CaCl_2_ with or without IL-1β for 2 h followed by addition of t-PA for 24 h (Scale bar = 10 mm, *n* = 3) (**A**). Effect of IL-1β on d-dimer concentration in thrombolytic blood clots (*n* = 3) (**B**). Effect of IL-1β on percentage of weight loss in thrombolytic blood clots (*n* = 3) (**C**). In panel A, significantly visual changes occurred between four groups, indicating that the weight losses of clots from IL-1β treatment groups (50 and 500 pg/mL) were less than that of the control group (0 pg/mL) (*n* = 3). *p ≤ 0.05, **p ≤ 0.01.

Thrombolytic activity in blood clot construct with or without IL-1β was investigated by measuring the amount of d-dimer (Fig. 5A–C). We found there were significantly visual variations amongst the four groups (Fig. 5A), which suggested that the fibrin networks under the influence of IL-1β and t-PA were susceptible to fibrinolysis compared to those in PBS alone. Consistent with this, after t-PA was added to the clots, there was a significant increase of d-dimer detected at every time point (1, 4, 8, 18, and 24 h), compared to the PBS/blood clot group (spontaneous fibrinolysis). Already within 1 h after the addition of t-PA there was an increased amount of d-dimer detectable, implying that the thrombolytic activity had been initiated (Fig. 5B). When IL-1β was added, there was a reduced rate of thrombolysis compared with IL-1β free controls (*p* < 0.05) (Fig. 5B). In addition, we also recorded a 10% weight loss due to spontaneous fibrinolysis in the PBS only group compared to the 500 pg/ml IL-1β treatment group (*p* < 0.01) (Fig. 5C).

### GSNO influences *in vivo* blood clot structure and large bone defect healing

**Figure 6:**
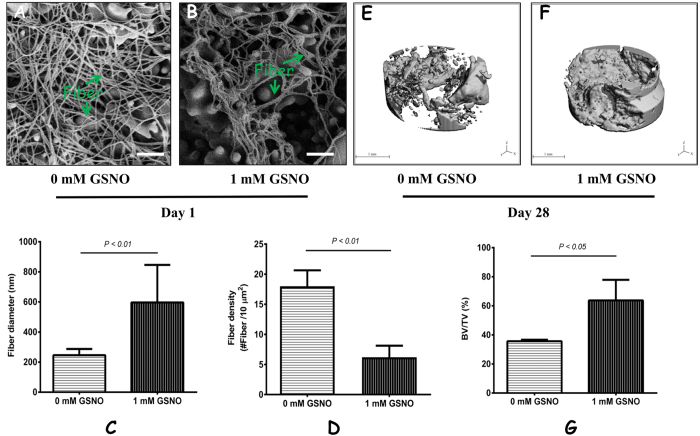
GSNO influences *in vivo* blood clot structure and large bone defect healing. A comparison of the morphologies of *in vivo* blood clot within delayed bone healing defects in the absence and presence of GSNO at day 1 using SEM (scale bar = 2 μm) (*n* = 3) (**A**,**B**). Fiber thickness and density were statistically analysed (**C**,**D**). Micro-CT revealed delayed bone defect healing with or without GSNO at day 28 (scale bar = 1 mm) (**E**,**F**) and BV/TV data were acquired (**G**) (*n* = 3).

Using SEM analysis, we found that the hematoma structures in large bone defects were altered as the result of 1 mM GSNO (Fig. 6A,B). The mean fiber diameter in the 1 mM GSNO treatment group was of 596.6 ± 249.4 nm compared to the controls at 245.8 ± 41.7 nm (*p* < 0.01) (Fig. 6C). By contrast, the fiber density (described as the fiber number per 10 μm^2^) in the 1 mM GSNO treatment group was significantly lower compared to the controls at 6.2 ± 1.9 vs. 18.0 ± 2.6, respectively (*p* < 0.01) (Fig. 6D). Micro-CT scanning showed there was a higher rate of bone formation at day 28 in the GSNO treatment group compared with the controls (Fig. 6E,F). The BV/TV of the control group was 38.64% ± 6.43%, whereas the ratio for 1 mM GSNO treatment group was 63.72% ± 14.13% (Fig. 6G), a difference that was statistically significant (*p* < 0.05).

## Discussion

The treatment of post-traumatic complications resulting from large bone defects, such as delayed bone union, represents a major challenge for orthopaedic surgeons^24^. Bone graft materials available have been used to improve bone regeneration, but many are fraught with complications, such as foreign body reaction, which results in fibrotic encapsulation^25^. The promise of stem cell treatments, using mesenchymally-derived cells, are still far from being realised for clinical use^26^. When a fracture occurs, extravasated blood from ruptured vessels quickly coagulates to produce a fracture hematoma as the first bridge between the broken bone fragments^27^. The hematoma provides little or no mechanical support and serves principally as a loose fibrin scaffold into which cells migrating from surrounding tissues (periosteum) can embed to exploit their angiogenic and osteogenic repertoire^28^. This is a comparatively rapid process that yields a mechanically stable hematoma composed mainly of platelets and fibrin fibers^29^. Parallel with the recruitment of platelets, enormous molecules, such as fibrinogen and proinflammatory cytokines, are released from the storage granules of activated platelets and further participate in the blood coagulation, thus modulating the clot structures^30^,^31^. Importantly, the simultaneous activation of coagulation reaction and inflammation have been shown to interact and engage in mutual crosstalk^32^. The structural properties of the fibrin matrices can influence cellular activity during the healing process^14^,^15^ but, surprisingly, few studies have investigated the effect of inflammatory cytokines on the structural properties of blood clots.

This study provides evidence of the morphological differences between hematomas from the normal bone healing defect and delayed bone healing defect. We found significantly higher endogenous levels of IL-1β in hematomas from the delayed healing defects, an indication that IL-1β may have a direct influence on the hematoma structures. We subsequently investigated the *in vitro* effects of IL-1β on fibrin polymerization and found that clots treated with 500 pg/mL IL-1β had shorter splint point, R, and K time, while the α angle and MA value were greater. These findings substantiated the hypothesis that a higher concentration of IL-1β speeds up coagulation, partly by facilitating the γ–γ cross-linking of fibrinogen between protofibrils^19^. Using high magnification SEM, we observed a notable decrease of fiber thickness and increased fiber density in the 500 pg/mL IL-1β group, which was strong evidence for the structure altering effects of IL-1β on blood clots. These changes to the fibrin structure resulted in significant changes of the clots’ mechanical properties. The Young’s modulus of blood clots in the 500 pg/mL IL-1β treatment groups was significantly higher compared to the control group and IL-1β treatment also resulted in a slower rate of thrombolysis. These results agreed with studies that reported decreased clot permeability resulting from inflammatory cytokines^33^,^34^. Our experiments demonstrate for the first time a direct association between the proinflammatory cytokine IL-1β and fibrin structure in human blood. IL-1β contains a β-barrel structural feature similar to fibroblast growth factor-2 (FGF-2). This β-barrel binds to the carboxyl terminus of the γ chain in fibrinogen, which is associated with fibrin polymerization, crosslinking, and platelet interaction^19^,^35^,^36^. The presence of IL-1β may also enhance the procoagulation activity of platelets by increasing the level of thrombin, somehow yielding clots with thinner fibers^37^,^38^. Exactly how IL-1β interacts with target cells at the fracture site is still unclear and requires further investigations.

GSNO is an antithrombotic that can effectively alter hemostasis by suppressing platelet activation and increase fiber diameter^20^,^39^. Intragingival application of GSNO in the rat can induce bone mineralization^40^, however, the mechanism for this is poorly understood. In the present study, we were able to demonstrate a substantial effect from GSNO on blood clot structure at a concentration of 1 mM, resulting in increased fiber diameter and decreased fiber density, which was associated with enhanced osteogenesis in the large bone defect model. This indicates that manipulation of fibrin clot architecture could be a viable means of improving bone healing.

The earliest event of fracture healing is the formation of a fibrin hematoma, in which the action of thrombin, produced by disturbances of blood vessels, instantaneously converts fibrinogen to produce fibrin monomers, which rapidly polymerize^41^. A range of cytokines, such as proinflammatory mediators, are known to be important for hemostasis in early stages of fracture repair^42^. However, the mechanisms that link proinflammatory cytokines, such as IL-1β and fiber structure is still unclear. The observations made in this study confirm the strong pro-coagulatory effects of IL-1β during thrombogenesis. These findings will aid in developing innovative approaches to modify hematoma structures, particularly the use of biological agents, which could assist in the regeneration of large segmental bone defects. The limitation of this study is species differences between human and rat blood plasma, the latter which is more hypercoagulable^43^,^44^. Therefore, a comparative study is needed of clots generated in human and a rat bloods in the presence of IL-1β.

There was evidence for species-specific differences in blood clot formation between bloods from humans and rats. Human whole blood was used for the *in vitro* study of the effect of IL-1β on clot formation, whereas the *in vivo* effects of GSNO on clot structure and bone healing was performed in rats. It is quite possible that differences in clot structure, such as fibrin size and clot density, could be due to interspecies differences. The control group in the IL-1β study was clots formed from 100 μL human whole blood and 0.2 M CaCl_2_ in the absence of IL-1β, whereas in the *in vivo* GSNO study, blood clot were allowed to form without any intervention in the bone defect. Rat blood is naturally hypercoagulable compared with human blood, an indicating of more active clotting factors, such as fibrinogen, in rats. Higher serum concentrations of fibrinogen leads to the formation of denser blood clots^45^. This reasoning may, therefore, explain the higher number and more densely packed fibers observed in rat blood (Fig. 6) compared with human blood (Fig. 3).

Overall, IL-1β has a profound effect of clot kinetics and alters the structural and biomechanical properties, as well as thrombolytic activity, of human blood clots. The observations made in this study give us a better understanding of the procoagulatory functions of IL-1β and supports the hypothesis that structural changes of the blood clot have measurable effects on bone healing, which suggest a possible mechanism for the delayed rate of healing in large bone defects. Additionally, pharmacological interventions, such as GSNO, when administrated directly into a large bone defect site, can alter the kinetics of fibre formation, yielding clots with thicker fibre size and decreased clot density. This approach could provide an innovative solution to the problems associated with delayed and non-unions bone defects.

## Methods

### *In vivo* study

#### Animal Surgery

**Figure 7:**
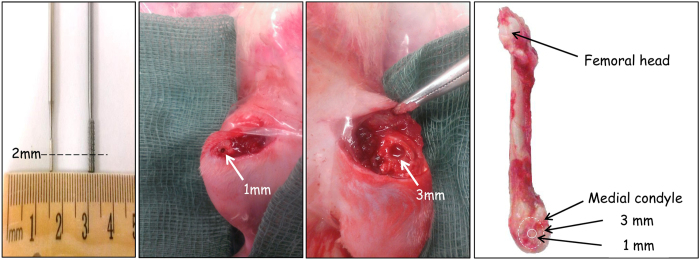
Establishment of normal bone healing and delayed bone healing defects. 1-mm- diameter and 3-mm-diameter defects (arrows) were created in the centre of medial condyle of rat bi-lateral femurs using Busch cross cut burs with cylinder shapes. To keep the consistency of created defects depths, a mark at 2 mm (dash line) was made on the surface of burs.

A total of 24 Fisher rats (6–8 weeks, 250 g) were used in these experiments. The small bone defects, which represented normal bone healing, were created by drilling a 1 mm diameter × 2 mm deep hole (Australian Jewellers Supplies Pty Ltd., Australia) in the femoral median condyle. The larger defect, representing the delayed bone healing, was made on the opposite femur of the animal and was 3 mm diameter × 2 mm deep (Fig. 7). The rats were administrated gentamycin (5 mg/kg) to prevent infection and euthanized at either 1 or 28 days post-surgery. All animal procedures were approved by the QUT Animal Ethic Committee (UAEC NO. 1400000023). All the methods were carried out in accordance with relevant guidelines and regulations.

#### Scanning electron microscopy (SEM)

A group of 6 rats were euthanized 1 day following surgery and the 1 and 3 mm hematomas harvested and thoroughly rinsed in phosphate buffer saline (PBS). The specimens were fixed in 3% glutaraldehyde overnight and the edge of hematoma structures studied using a TM3000 SEM (Hitachi High-Technologies Corporation, Japan), since hematoma edges have a direct contact with the boundaries of bone defects. SEM images were acquired at 50× and 1000× magnification at high voltage (HV) range of 15 KV. Fibers were selected at random for analysis.

#### Microtomograhy and Histology

Twenty-eight days post-surgery, 6 rats were euthanized and the bone defects harvested and fixed in 4% paraformaldehyde (PFA) (pH = 7.4) overnight at 4 °C. Radiographs were taken using a Micro-CT scan (μCT 40, Scanco, Brüttisellen Switzerland) to evaluate bone formation. The specimens were subsequently de-mineralized in 10% ethylene-diamine-tetraacetic acid (EDTA) solution (pH = 7.4) for 4 weeks then embedded in paraffin. Sections were cut to 5-μm thickness with a microtome (Leica Microsystem, Nussloch, Germany), de-waxed and rehydrated, then stained with haematoxylin and eosin (H&E). Images of the samples were captured with a Leica SCN400 Slider Scanner (Leica Microsystem, Germany)^46^.

#### Enzyme-Linked Immuno-Sorbent Assay (ELISA)

Six animals were euthanized on day 1 post-surgery and the hematoma samples within the 1 and 3 mm bone defects harvested and lysed in RIPA buffer (Sigma-Aldrich Corp., St Louis, MO). The lysates were centrifuged for 5 mins at 5,000 × *g* at 4 °C, and the supernatants transferred to new tubes. Protein concentrations were measured using a Bicinchoninic Acid (BCA) Protein Assay (Pierce, Thermo Fisher Scientific) and IL-1β proteins were analysed by chemiluminescence-based ELISA kit (R&D Systems, Inc., Minneapolis, MN) following the manufacturer’s instructions. At least six replicates were assayed from each group^47^.

### *In vitro* study

#### Reagents

Human Alpha Thrombin (HT 1002a) and human plasminogen (HPg 2001) were acquired from Enzyme Research Laboratories (Bulimba, Australia). Human Interleukin-1β was purchased from Invitrogen Thermo Fisher Scientific Inc (Victoria, Australia). Recombinant human tissue-type plasminogen activator (t-PA) and d-dimer (D2D) ELISA Kit were purchased from Antibodies-Online Inc. (Atlanta, USA). S-nitrosoglutathione (GSNO) was ordered from Sigma Aldrich (Sydney, NSW, Australia).

#### Blood samples

Human whole blood samples were sourced from the Australian Red Cross Blood Bank. The plasma fibrinogen concentrations measured by the Clauss method ranged from 2 to 2.5 g/L^48^. All blood samples were collected from healthy donors who had no history of coagulation disorders or had taken any haematological related medication in the preceding 6 months. Ethical approval (1500000918) was granted by the University Human Research Ethics Committee (UHREC) at Queensland University of Technology. Informed consent was obtained from all subjects and all the methods were carried out in accordance with relevant guidelines and regulations.

#### Thromboelastography (TEG)

Thromboelastography was performed using a TEG^®^ 5000 Series Hemostasis Analyser System (Hemoscope Corporation, Niles, IL) to measure the viscoelastic properties of citrated whole blood clots. Three-hundred and twenty microliters of human blood were pipetted into oscillating plastic TEG cups and the coagulation process was re-calcified by the addition of 20 μL of 0.2 M calcium chloride (CaCl_2_) solution with or without HEPES (4-(2-hydroxyethyl)-1-piperazineethanesulfonic acid) buffered IL-1β (50 or 500 pg/mL). The profile of clot formation was traced using the TEG5000 Series Hemostasis System for 1.5 h at 37 °C. The hemostatic parameters, such as split point (SP), reaction time (R-time), coagulation time (K-time), alpha angle (α), and maximum amplitude (MA), were recorded and analysed. SP reflects the initial fibrin formation, R time measures the initial clot formation time, both K time and α angle are direct indicators of thrombosis formation speed, and MA is indicative of the maximal strength of the clots^49^,^50^. The procedure was repeated three times.

#### Scanning electron microscopy

Blood clots were prepared from 100 μL of whole blood with 10 μL of 0.2 M CaCl_2_ with and without IL-1β (50 or 500 pg/mL). These mixtures were incubated at 37 °C for 2 h to allow clotting to proceed and then fixed in 3% glutaraldehyde overnight. The resulting clots were rinsed with PBS at least 3 times and dehydrated in an ascending alcohol series (50%, 70%, 90%, and 100%) and dried in a CO_2_ critical point drying apparatus. The specimens were then mounted on aluminium stubs and coated with gold-palladium, and micrographs captured by SEM (FEI, USA). Representative images were captured at 5,000× magnification and the images analysed using a method described previously^51^,^52^. Fiber thickness and density were assessed from SEM images using image analysis software package Image J (version 1.43). Fiber diameter (*n* = 90) from each concentration was analysed and reported as an average. Three images were analysed from each clot sample and data were acquired from each of the three clots from each concentration.

#### Compressive moduli

Blood clot stiffness was quantified with an Instron 5848 Microtester (Canton, MA) using the method established by Davis *et al*. and Textor *et al*.^53^,^54^. Blood clots with or without IL-1β (50 or 500 pg/mL) were allowed to polymerize in a 24-well plate for 24 h at 37 °C. The length and diameter of the blood clots were recorded and clots incubated in PBS for 1 h prior to stiffness measurements taken. Blood clots were loaded on a flat plate and compressed at 10 mm/min with a preload of 5 N with an extension limit of 3.5 mm. The linear region of force-displacement curve was determined within strain ranging from 0 to 5%, and the slope was defined as Young’s modulus (compressive stiffness), which were calculated using equations 1, 2, 3. The experiment was repeated three times.


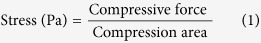



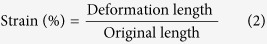



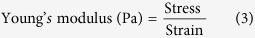


#### Thrombolytic assay

The thrombolytic activities of whole blood clots were assessed in a suspended clot system using the method detailed by Shiu *et al*.^55^. Hundred microliters of 0.2 M CaCl_2_ with or without IL-1β (50 or 500 pg/mL) was added to 1 mL whole blood and incubated for 2 h at 37 °C. Once the blood clotted, the clots were transferred into new vials containing a final concentration of 5.4 μg/mL human Glu-plasminogen in 3 mL of PBS buffer suspension. The thrombolytic reaction of the clots was activated by addition of 0.25 μg/mL t-PA and agitated gently at 37 °C. Aliquots were removed at nominated time points (1, 4, 8, 18, and 24 h) and spun at 1000 × *g* for 3 min. Supernatants (100 μL) were assayed using a d-dimer ELISA kit (Antibodies-Online Inc., USA). The quantity of d-dimer, released from the clots, reflected the thrombolytic activity of the samples. Additionally, fibrinolysis was measured in blood clots in PBS without t-PA to account for spontaneous fibrinolysis and was defined as controls. The weight of clotted bloods was also recorded at 0, 18, and 24 h to assess clot weight change. The experiment was repeated three times.

### *In vivo* study

#### Scanning electron microscopy & Microtomograhy

Large delayed healing bone defects were induced in the bi-lateral femoral condyles of 6 rats as described above. GSNO at 1 mM was thoroughly mixed with blood within the defects in the left condyle of rats and allowed to completely clot before closing the incision. The defects in the right condyle of the same animals were left empty and defined as the control groups. Three rats were euthanized at day 1 post-surgery and the harvested hematomas were processed and analysed by SEM (FEI, USA) at 10,000× magnification. Measurements of fiber diameter and density at the edge of hematomas were made randomly and analysed for statistical significance. The remaining 3 rats were euthanized at day 28 post-surgery and *de novo* bone tissues within the defects assessed using μCT scanner as described above. Samples were used for SEM and μCT assay in triplicate, respectively.

### Statistical analyses

Statistical differences were assessed between two groups using unpaired two-tailed Student t-tests. A one-way analysis of variance (ANOVA) with Holm-Sidak’s tests was performed from GraphPad Prism 6.0 for multiple comparisons. Data from all the experiments were expressed as averages plus and minus standard deviation (SD). A *p* value of less than 0.05 was determined as a significant difference.

## Additional Information

**How to cite this article**: Wang, X. *et al*. Alteration of blood clot structures by interleukin-1 beta in association with bone defects healing. *Sci. Rep.* **6**, 35645; doi: 10.1038/srep35645 (2016).
